# Study on the Effect of Inter-Layer Cooling Time on Porosity and Melt Pool in Inconel 718 Components Processed by Laser Powder Bed Fusion

**DOI:** 10.3390/ma16113920

**Published:** 2023-05-24

**Authors:** Niccolò Baldi, Alessandro Giorgetti, Marco Palladino, Iacopo Giovannetti, Gabriele Arcidiacono, Paolo Citti

**Affiliations:** 1 Department of Engineering Science, Guglielmo Marconi University, 00193 Rome, Italy; n.baldi@unimarconi.it (N.B.); g.arcidiacono@unimarconi.it (G.A.); p.citti@unimarconi.it (P.C.); 2 Department of Industrial, Electronic and Mechanical Engineering, Roma Tre University, 00146 Rome, Italy; 3 Baker Hughes—Nuovo Pignone, 50127 Florence, Italy; marco.palladino@bakerhughes.com (M.P.); iacopo.giovannetti@bakerhughes.com (I.G.)

**Keywords:** laser powder bed fusion, inter-layer cooling time, melt pool morphology, nickel-based superalloys, Inconel 718, design for additive manufacturing

## Abstract

This paper investigates the effects on the material microstructure of varying the Inter-Layer Cooling Time (ILCT) during the printing process in laser powder bed fusion (L-PBF) multi-laser machines. Despite these machines allowing higher productivity rates compared to single laser machines, they are affected by lower ILCT values, which could be critical for material printability and microstructure. The ILCT values depend both on the process parameter sets and design choices for the parts and play an important role in the Design for Additive Manufacturing approach in L-PBF process. In order to identify the critical range of ILCT for this working condition, an experimental campaign is presented on the nickel-based superalloy Inconel 718, which is widely used for the printing of turbomachinery components. The effect of ILCT on the microstructure of the material is evaluated in terms of porosity and melt pool analysis on printed cylinder specimens, considering ILCT decreasing and increasing in the range of 22 to 2 s. The experimental campaign shows that an ILCT of less than 6 s introduces criticality in the material microstructure. In particular, at an ILCT value of 2 s, widespread keyhole porosity (close to 1‰) and critical and deeper melt pool (about 200 microns depth) are measured. This variation in melt pool shape indicates a change in the powder melting regime and, consequently, modifications of the printability window promoting the expansion of the keyhole region. In addition, specimens with geometry obstructing the heat flow have been studied using the critical ILCT value (2 s) to evaluate the effect of the surface-to-volume ratio. The results show an enhancement of the porosity value (about 3‰), while this effect is limited for the depth of the melt pool.

## 1. Introduction

Additive manufacturing (AM), contrary to the conventional machining methods in which the product is manufactured by removing the unwanted material from the blank [1], allows structure and component fabrications in a layer-by-layer material deposition directly from a sliced CAD model [1,2,3,4,5,6,7].

Laser powder bed fusion (L-PBF), according to ISO/ASTM 52900 standard, is an AM process in which thermal energy selectively fuses regions of a powder bed. This technology is revolutionizing the manufacturing approach to obtain near-net-shape components, especially for products with high geometric complexity [8,9,10]. The main competitive advantages of L-PBF compared to other metal AM technologies are the low heat generation and resulting lower distortion and, consequently, the high accuracy of the finished part, which can be produced at a relatively high production speed and low material cost. Overall, this technique can be considered as probably the most widespread, versatile and promising among the metal AM techniques [11]. Due to these advantages, the L-PBF process can be used to produce geometrically complex, near-net-shape parts with good surface integrity and superlative mechanical behavior [12]. In addition, L-PBF can process many metal alloys, such as nickel-based superalloys, which are extremely important for high-value engineering products. The use of L-PBF can solve some of the manufacturing problems of these materials, which are difficult to manufacture using conventional machining methods due to their high hardness and low thermal conductivity.

Despite all these advantages compared to the conventional machining methods, this process is affected by low efficiency in terms of material handling, high maintenance costs and low production volume [13]. The build rate of L-PBF is roughly estimated to be around 40 cm^3^/h, lower than that estimated for electron beam melting (EBM) and direct energy deposition (DED) of 70–100 and 140 cm^3^/h, respectively [14].

As a result, the need to make this process more productive and competitive with other technologies is becoming the most crucial goal in this field.

Increasing productivity [15,16] means raising the amount of volume deposed and melted in the unit of time; this goal is achievable through the implementation of the following solutions:

- Process parameters optimization [17,18] using high layer thickness [15], fast scanning speed and increasing hatch distance [19].
- New printing strategy as Hull-Core [16].
- Increasing the number of heat sources using multi-laser machines [20].

Among all the proposed solutions, using multi-laser machines is the best, allowing print volumes for the job to be doubled or even tripled.

Although these machines allow sharp increases in print volumes, they suffer from very low Inter-Layer Cooling Time (ILCT) [21,22,23].

**Figure 1:**
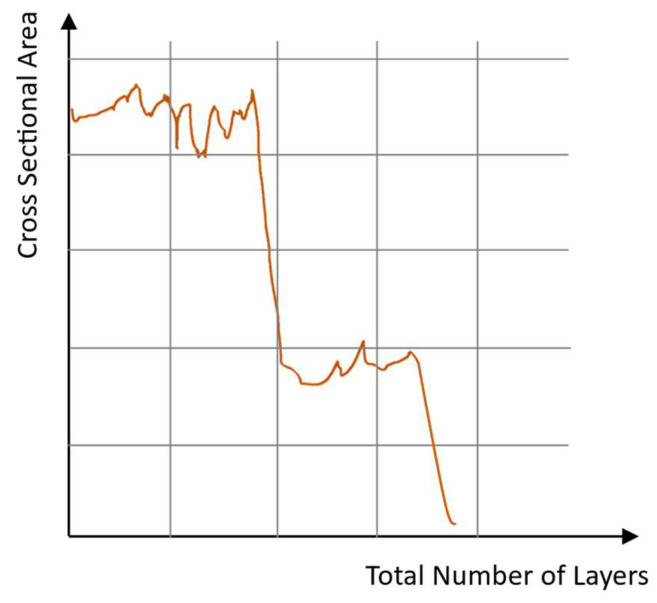
An example of a section and corresponding ILCT variation during the printing of a component.

The ILCT is calculated as the time between the exposure of one layer and the next, so it is different from the Inter-Layer Time (ILT) [22] constant during the printing when no laser exposure is activated, which is also calculated considering the time required for powder recoating. The length of this time between successive laser scans of a point on the cross-section varies from layer to layer, so this time is not constant across all layers due to geometry variations in the cross-sectional areas per layer (see Figure 1). This parameter influences the average input rate into the part and changes the temperature of the substrate.

Since the process is essentially thermally driven, the difference in thermal history between layer parts or from layer to layer could affect the mechanical properties and microstructure [24,25,26,27,28,29,30,31,32,33,34,35,36,37,38]. According to Williams et al. [21], this phenomenon has not been extensively studied in the literature for the L-PBF process; only a few studies have been performed for the Ti-6Al-4V alloy [39,40]. On the contrary, some effects of ILCT in DED have been reported [41,42,43] and the correlation between these parameters and porosity, Vickers hardness and compressive strength is identified. Moreover, it is pointed out in [44] that ILCT is a less important parameter in L-PBF due to the different thermal and temporal conditions between the two processes, which is mainly due to the shorter ILCT in L-PBF.

As previously mentioned, the variation of this parameter becomes predominantly critical on multi-laser machines where the exposure times for each layer are considerably shorter than in single laser printers as multiple lasers work in parallel on the same printing platform.

**Figure 2:**
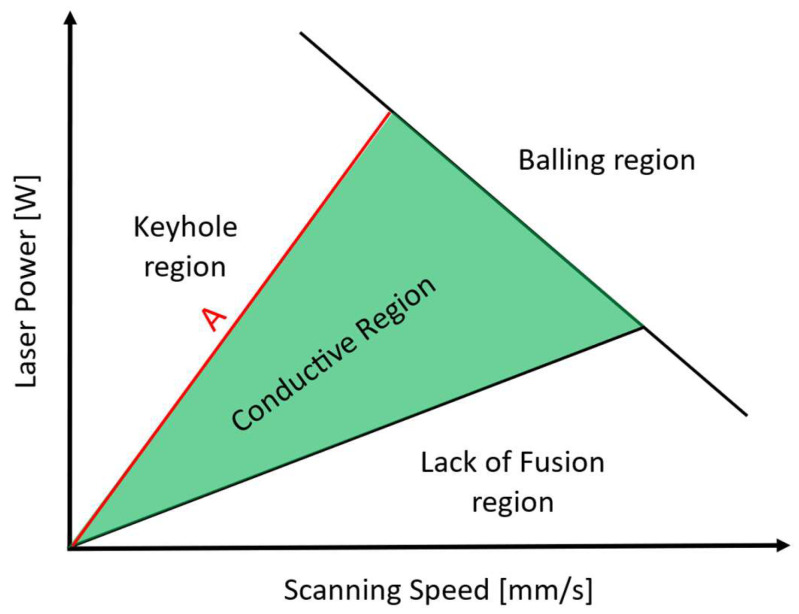
Printability window in laser power and scanning speed space, the red line separates the conductive region from the keyhole one.

In multi-laser printers, a value of ILCT equal to a few seconds can be reached, hypothetically increasing the substrate temperature. So, what is theoretically expected is a variation of the material’s printability window (Figure 2 [18,45,46,47]) working in these particular thermal conditions.

In previous literature studies [46,48,49], it has been observed that the change in the melting regime of the powder, due to an increase in substrate temperature, promotes the transition of the melting regime from conductive mode to keyhole mode. In this condition, the heat transfer and melt pool formation are completely governed by convection; the melt pool tends to be deeper and deeper, assuming the typical keyhole shape. Moreover, the keyhole mode melting regime can promote the formation of gas bubbles entrapped inside the melt pool due to the vaporization of low melting point elements within the alloy [50,51].

This results in the widespread formation of spherical porosity that can affect the density of the material.

This phenomenon turns out to be of considerable importance during the development of set process parameters [52] that will have to take into account the possible variation in component temperature and be sufficiently robust against it, so the identification of printability has to be a function of the consolidated substrate’s temperature. On the other hand, this represents an important constraint during the design of a component in order to avoid reaching the component’s temperatures far from the conditions of stability of the melt pool.

In [21,23], it has been assessed for 316L stainless steel how the variation of ILCT affects the substrate temperature, material density, microstructure and hardness; despite this, the use of a single laser machine during testing does not allow the investigation of ILCT levels in the order of units of seconds.

In addition, the literature lacks studies on the effect of ILCT on nickel-based superalloys, widely used for printing components in the oil and gas industry and on melt pool shape in terms of width and depth. It is also important to underline that nickel-based superalloys show some similarity in printability properties in the L-PBF process [53].

This paper investigates the effects of ILCT variations on the microstructure of IN718 superalloys processed by a multi-laser machine to obtain an ILCT value of a few seconds. The material microstructure will be evaluated through porosity and melt pool analysis by printing single tracks on powder bed, as suggested in the literature [11,52,53,54,55,56,57].

The paper has the following structure: Section 2 describes the experimental procedure with a specific focus on the specimen’s geometry, ILCT levels and process parameter set. Section 3 presents the porosity and melt pool analysis results obtained from the experimental tests on Inconel 718 alloy specimens. Section 4 discusses the effect of ILCT variation on the material microstructure. Finally, the Conclusions summarizes the results and provides concluding remarks.

## 2. Materials and Methods

The specimens used in this case study were made in Inconel 718, one of the most common nickel-based superalloys with which to build components in additive manufacturing.

The main characteristics of Inconel 718 are shown in Table 1 (chemical composition) and Table 2 (mechanical and thermal characteristics) [52,58].

The layer thickness used to build the specimens was 60 microns, with a particle size of powder between 20 and 63 microns, obtained through a gas atomization process.

The Renishaw AM500Q machine was provided with 4 ytterbium fiber lasers characterized by a beam wavelength of 1070 nm and a minimum spot size for the laser beam of 82 μm. A laser power of 500 W was the maximum value available for each laser. The building platform temperature was kept constant during all the printing at a temperature of 170 °C, while an argon gas flow was used to maintain oxygen content inside the building chamber under 100 ppm.

All available lasers provided in the machine were used during the printing to reproduce the printing conditions of components properly.

All the tested specimens were printed using a set of medium-high volumetric energy density (VED) process parameters chosen according to the results reported in the literature [59], as shown in Table 3.

The experiments were carried out in a high volumetric energy zone to study the effect of ILCT variation in the area close to the keyhole melting regime, where defect formation is promoted. This choice was made to understand whether a short ILCT could increase the tendency of defect formation or not. In addition, working in this VED region was interesting to avoid high residual stresses in the component as the remelting of several previous layers was obtainable.

The presented analysis of ILCT’s variation effect on material microstructure consisted of two main phases:

1. Investigation of ILCT’s variation effect on the material microstructure.
2. Evaluation of obstructing heat flux geometries on critical ILCT values.

### 2.1. Study of ILCT’s Variation Effect on Material Microstructure

This phase investigates if a critical level of ILCT negatively affects the material microstructure. The effect on the material microstructure was evaluated as a modification on porosity level and melt pool characteristic dimensions and shape ratio.

ILCT has been studied, considering the increasing and decreasing order, to reproduce the heating and cooling effect on a component due to geometry section variations. The range of ILCT investigated was from 22 s to 2 s in steps of 4 s.

**Figure 3:**
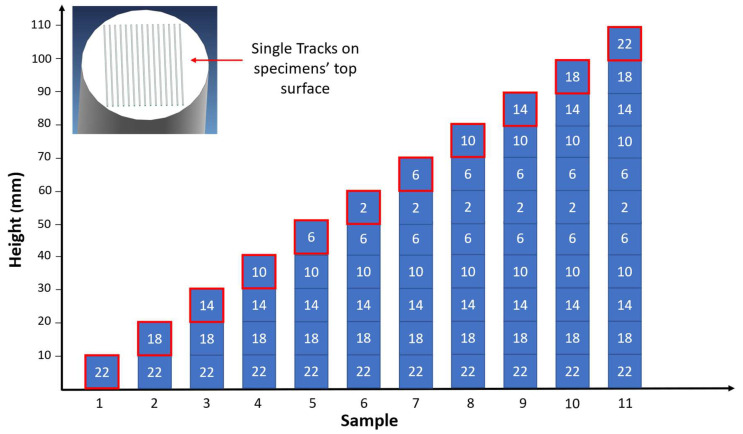
Overview of ILCT levels tested in each specimen, the red box indicates the analyzed section for each specimen. Image copyright (2023) Baker Hughes Company—All rights reserved. Used with permission.

A cylinder specimen was printed for each ILCT level tested, and each ILCT level was kept constant for ten millimeters in height. Thus, 11 specimens were made that were exposed to the ILCTs shown in Table 4. Consequently, the specimens had different heights, as shown in Figure 3. A second set of the same samples were printed for repeatability assessment.

Single tracks and single laser exposition on the powder bed were also made on the top surface of each specimen (Figure 3) in order to investigate the variation in melt pool shape and calculate the melting regime at different ILCT levels in relation to the melt pool dimensions ratio [46].

**Figure 4:**
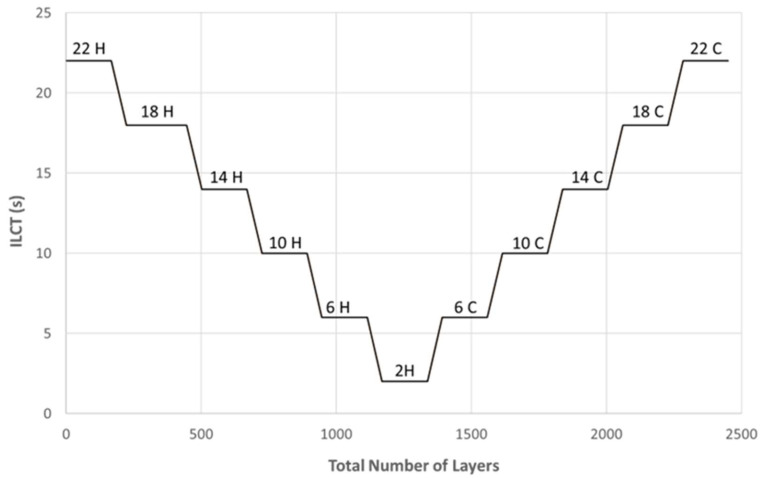
ILCT variation as a function of the total number of layers. “H” indicates the heating phase while “C” indicates the cooling phase.

Figure 4 shows the variation of ILCT as a function of the total number of layers, during the printing, for specimen 11 (sample code 22 C).

The planned ILCT for the specimens was obtained using customized ghost parts characterized by zero laser power exposition.

**Figure 5:**
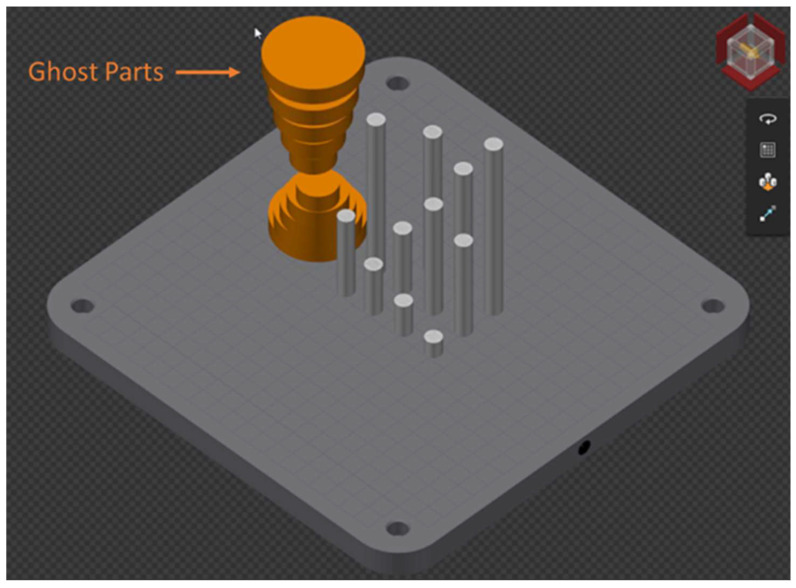
Specimens and ghost parts placement on the building platform. Image copyright (2023) Baker Hughes Company—All rights reserved. Used with permission.

Considering the process parameters and laser assignment, the build file was created using Renishaw Quantam Software Version 5.3.0.7105. The specimens were placed on the building platform, as shown in Figure 5.

**Figure 6:**
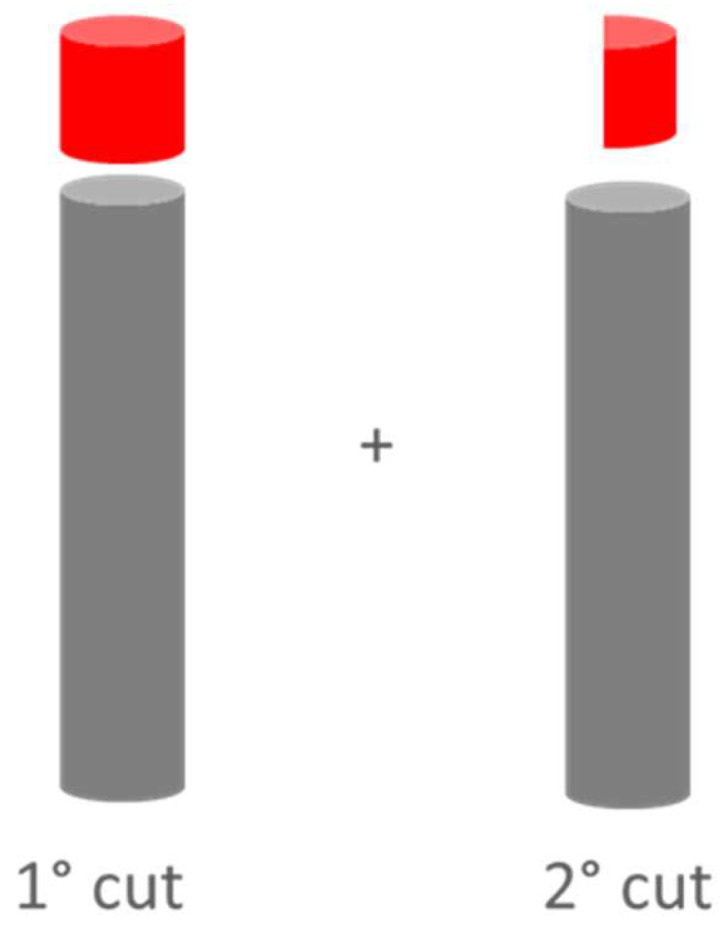
Cutting planes of specimens, the red sections indicate the section to be analyzed.

The printed specimens were detached from the building platform using a wire EDM machine (ECUT EU MS Genesi) and machined to obtain a mockup with a height of ten millimeters, starting from the top base. Then, the mockup was sectioned along an axial plane, as shown in Figure 6.

The specimens were then etched with oxalic acid to highlight the melt pool shape. The microstructural analyses were then carried out as follows:

- A porosity analysis was performed to estimate the densification level and the presence of defects in the material. This analysis was performed using a Leica Leitz DMRME (Leica Microsystems GmbH, Wetzlar, Germany) optical microscope, and the percent porosity was evaluated by analyzing six fields for each specimen. Then, the acquired images were post-processed using ImageJ 1.53 (National Institute of Health, Bethesda, MD, USA) software. In order to measure the porosity percentage, a thresholding image processing was applied to the acquired images.
- A melt pool analysis was performed on the specimens using the Leica Leitz DMRME (Leica Microsystems GmbH, Wetzlar, Germany), and then a chemical etching with oxalic acid was carried out to highlight the melt pool boundaries. For each analyzed configuration, five random single tracks were measured in terms of melt pool width and depth [52].

### 2.2. Effect of Obstructing Heat Flux Geometries on Material Microstructure for Critical ILCT

This phase aims to investigate whether a geometry that obstructs the heat flux criticality affects microstructure for the critical value of ILCT identified in the previous phase [23]. This analysis could be interesting because this condition can hypothetically promote the overheating of the component, resulting in increased keyhole porosity and a deeper melt pool.

**Figure 7:**
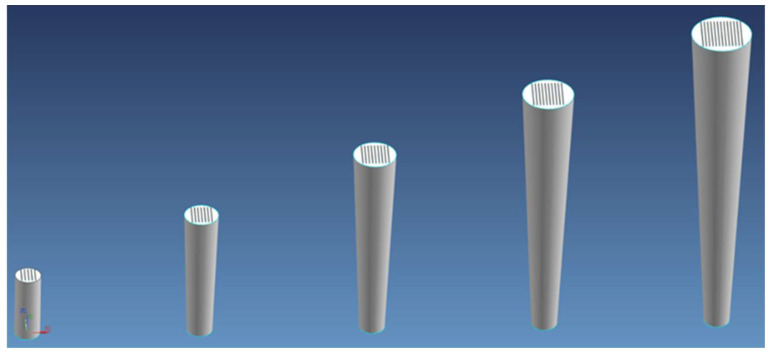
Specimen shapes. Image copyright (2023) Baker Hughes Company—All rights reserved. Used with permission.

The specimens were modelled as cylinders and inverted truncated cones in function of the area to be evaluated, as shown in Figure 7.

The usage of inverted truncated cone specimens was set to reproduce very hostile heat transfer conditions. This geometry could help the element to overheat progressively as each layer has a larger area to be exposed compared to the one below (assuming that the heat transfer along the component is predominantly governed by conduction through the below layer surface [60]). This effect will be higher as the height of the sample increases.

The dimensions and the investigated area for each specimen are listed in Table 5. A second set of the same samples was printed for repeatability assessment.

In order to assess and calculate the melting regime for each tested configuration, several single tracks were printed on the top surface of each specimen (Figure 7).

As in Section 2.1, the ILCT value was kept constant along the specimen’s height using customized ghost parts [18].

**Figure 8:**
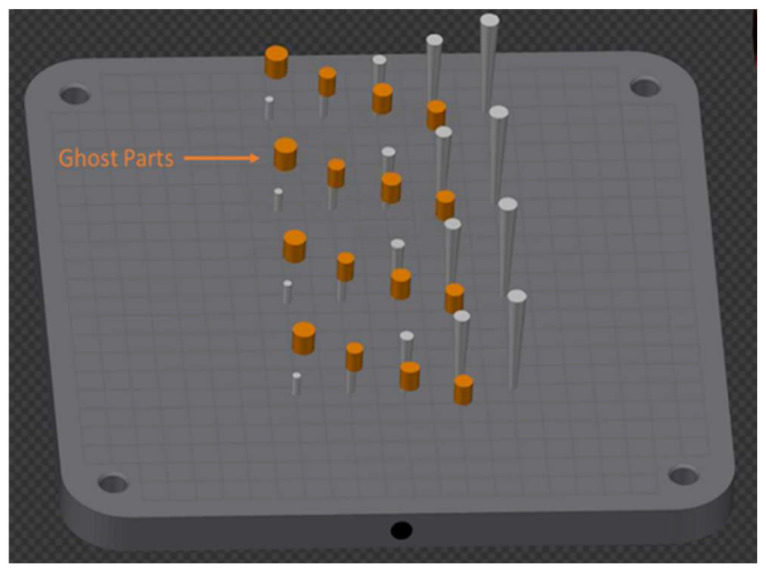
Specimens’ placement on the building platform during the job. Image copyright (2023) Baker Hughes Company—All rights reserved. Used with permission.

The build file was created following the procedure described in Section 2.1. The specimens were placed on the building platform, as shown in Figure 8. The preparation of the samples and the micrographic analysis followed the procedure described in Section 2.1.

## 3. Results

### 3.1. ILCT’s Variation Effect on Material Microstructure

**Figure 9:**
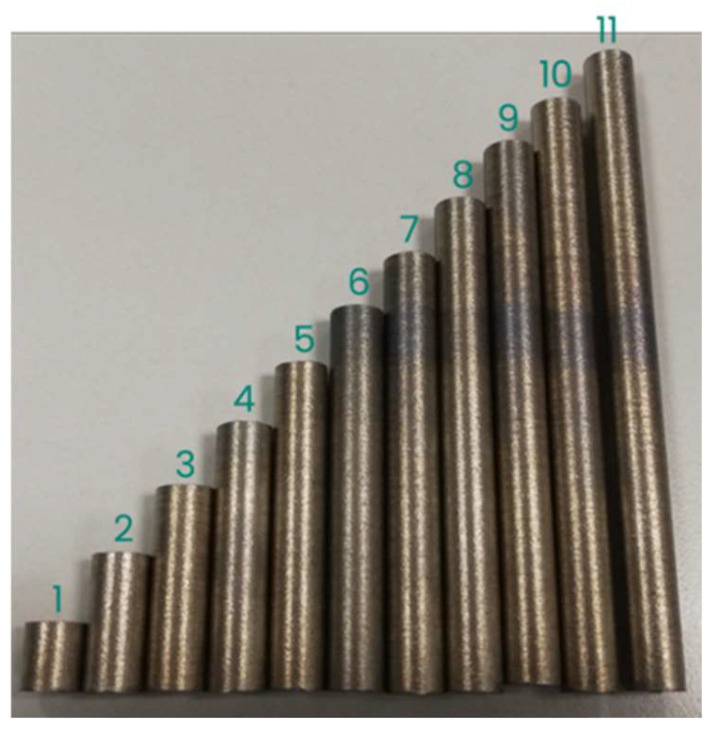
Specimens after detachment from the building platform. The numbers indicate the sample number as defined in Table 4. Image copyright (2023) Baker Hughes Company—All rights reserved. Used with permission.

**Figure 10:**
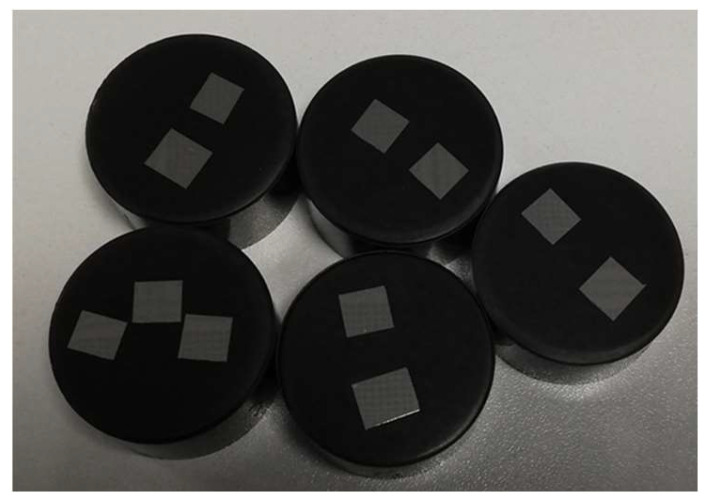
Specimens after cutting, embedding in conductive resin and polishing. Image copyright (2023) Baker Hughes Company—All rights reserved. Used with permission.

Figure 9 shows the specimens in the as-built condition after the detachment from the building platform and Figure 10 shows them after cutting, embedding in conductive resin and polishing.

**Figure 11:**
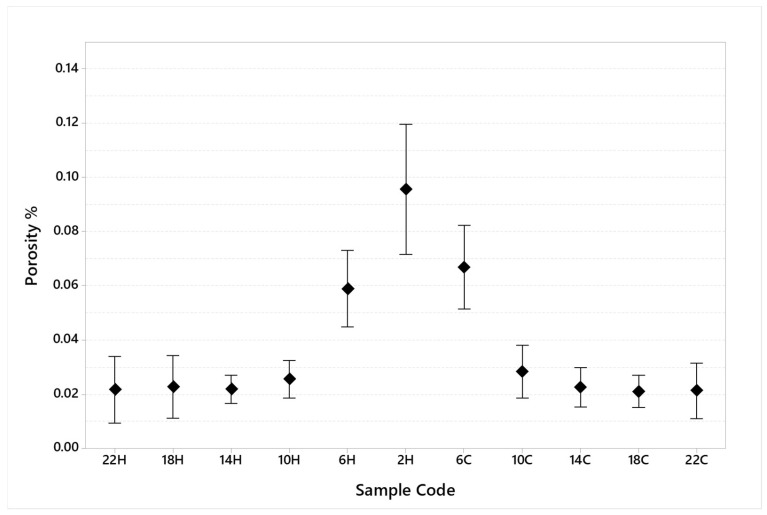
Porosity measured for each specimen (95% confidence interval).

The porosity results in terms of average and standard deviation for each specimen are summarized in Table 6 and plotted in Figure 11.

**Figure 12:**
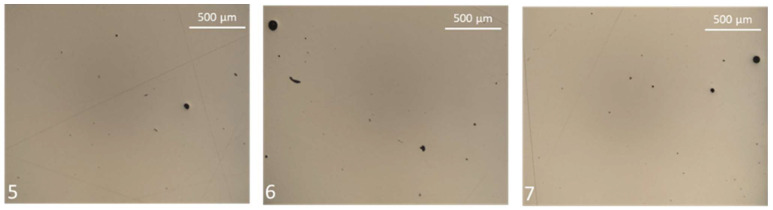
Evidence of Keyhole porosity in specimen number 5, 6 and 7. Image copyright (2023) Baker Hughes Company—All rights reserved. Used with permission.

Figure 12 shows the most representative images for the specimens identified as most porous, specimens 5, 6 and 7, respectively, while Table 7 tracks the maximum defect diameter measured for each specimen.

Table 8 shows the dimension of the melt pool in terms of width and depth and the width/depth ratio generally used to calculate whether or not the keyhole melting regime is present during the printing of specimens.

**Figure 13:**
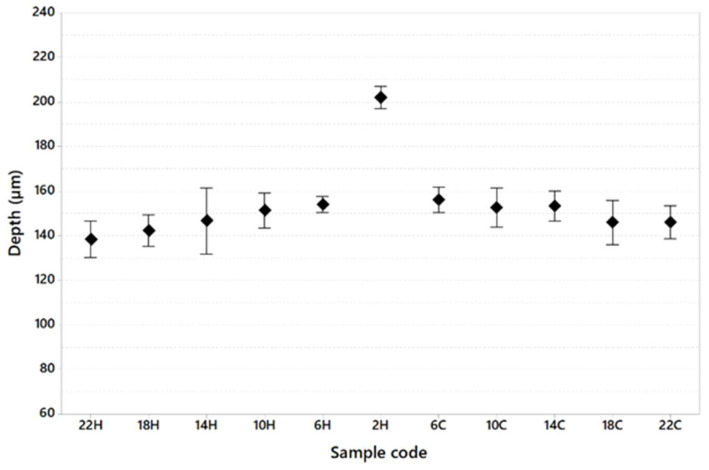
Melt pool depth as a function of sample code (95% confidence interval).

**Figure 14:**
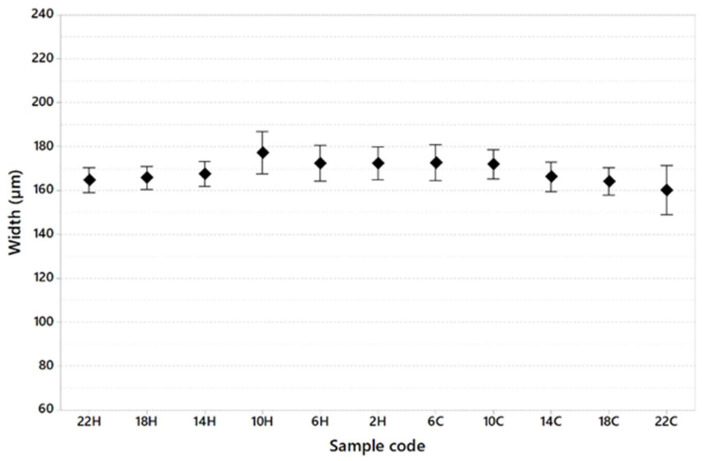
Melt pool width as a function of sample code (95% confidence interval).

**Figure 15:**
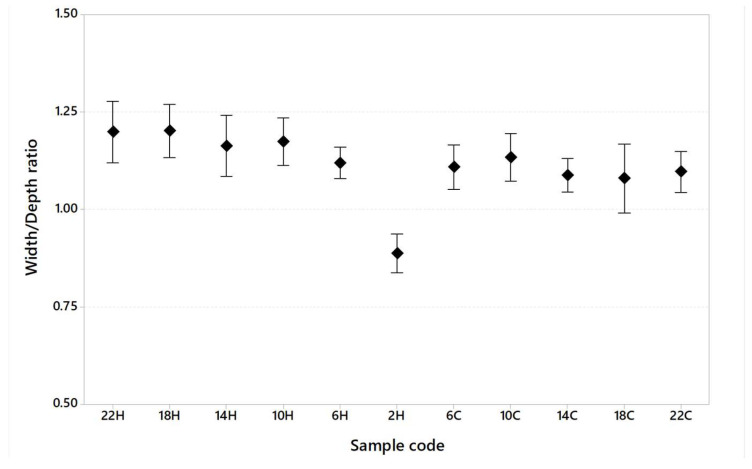
Width/depth ratio as a function of sample code (95% confidence interval).

**Figure 16:**
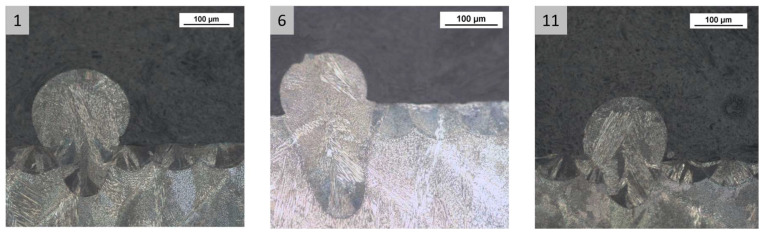
Melt pool shape: sample 1 ILCT = 22 s (heating); sample 6 ILCT = 2H (heating) seconds; sample 11 ILCT 22 s (cooling). Image copyright (2023) Baker Hughes Company—All rights reserved. Used with permission.

Figure 13, Figure 14 and Figure 15 show melt pool depth, melt pool width and width/depth ratio, respectively, for each specimen. Figure 16 shows the melt pool shape for configurations 1, 6 and 11.

### 3.2. Effect of Geometries Obstructing Heat Flux on Material Microstructure

**Figure 17:**
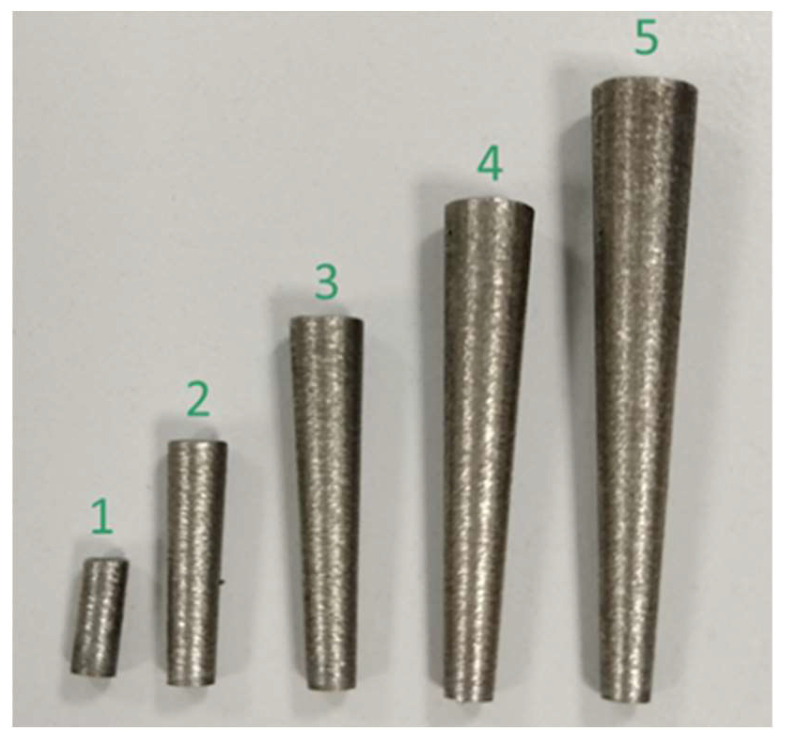
Specimens after detachment from the building platform. The numbers indicate the sample as defined in Table 9. Image copyright (2023) Baker Hughes Company—All rights reserved. Used with permission.

Figure 17 shows the specimens in the as-built condition after the detachment from the building platform.

**Figure 18:**
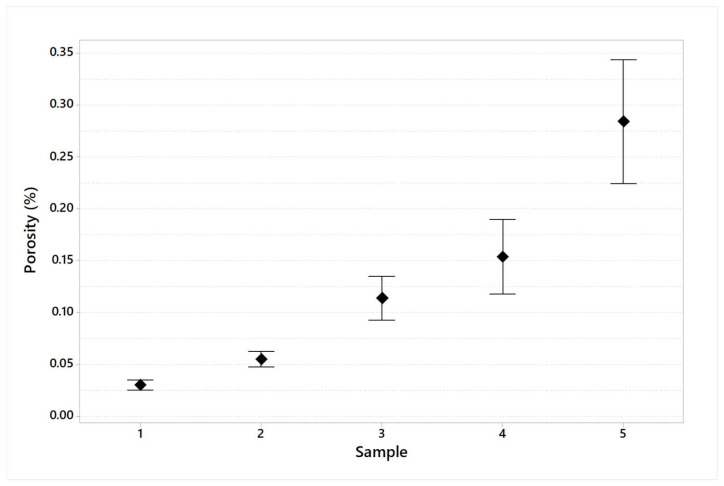
Porosity measured for each specimen (95% confidence interval).

The porosity average and standard deviation results for each specimen are tracked in Table 9 and plotted in Figure 18.

**Figure 19:**
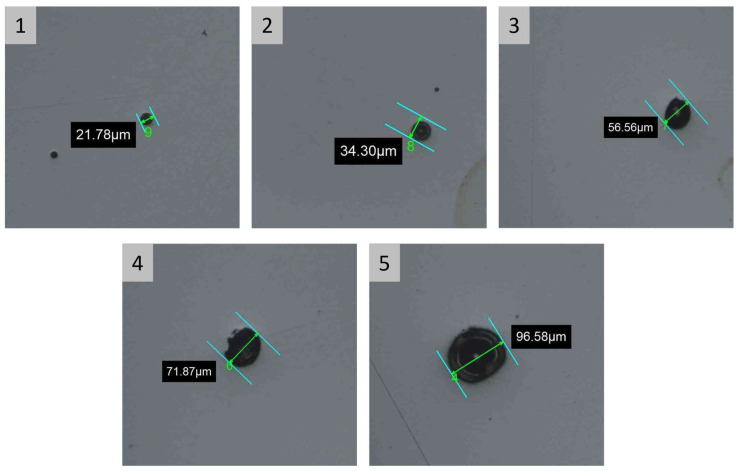
Maximum defect diameter detected in each specimen during porosity analysis, defect diameter increases as function of analyzed specimen. The number in the gray box indicates the specimen as defined in Table 9. Image copyright (2023) Baker Hughes Company—All rights reserved. Used with permission.

In order to evaluate the size of defects that affect the specimens’ microstructure, Figure 19 shows some images for the diameter of the maximum detected defect for each specimen, and the corresponding values are summarized in Table 10.

**Figure 20:**
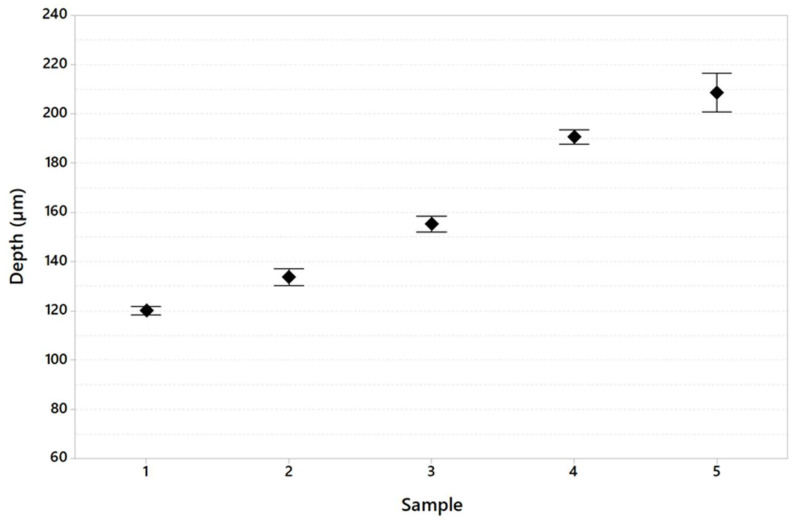
Melt pool depth variation in the specimen tested (95% confidence interval).

**Figure 21:**
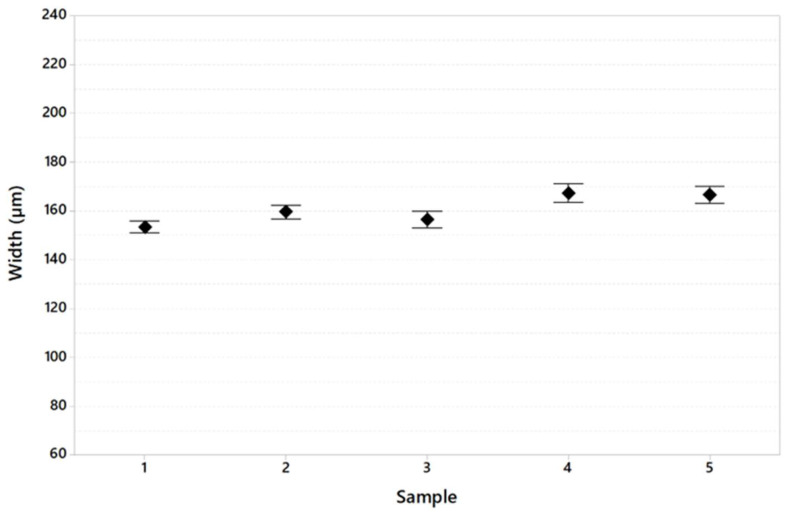
Melt pool width variation in the specimen tested (95% confidence interval).

**Figure 22:**
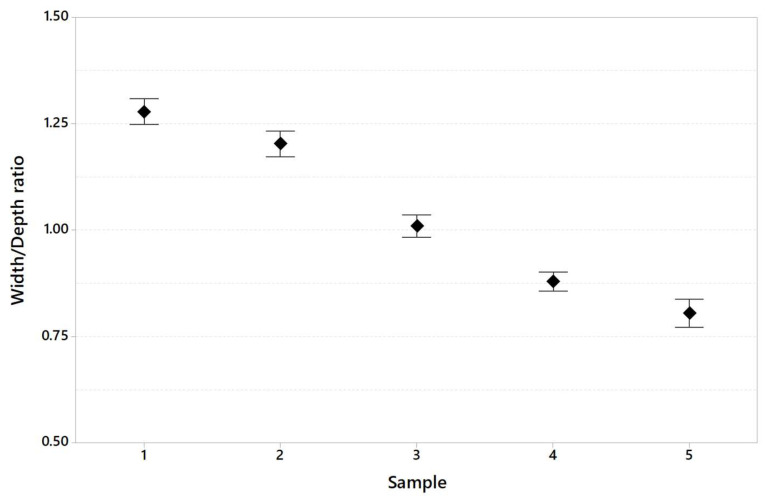
Width/Depth ratio variation in the specimen tested (95% confidence interval).

**Figure 23:**
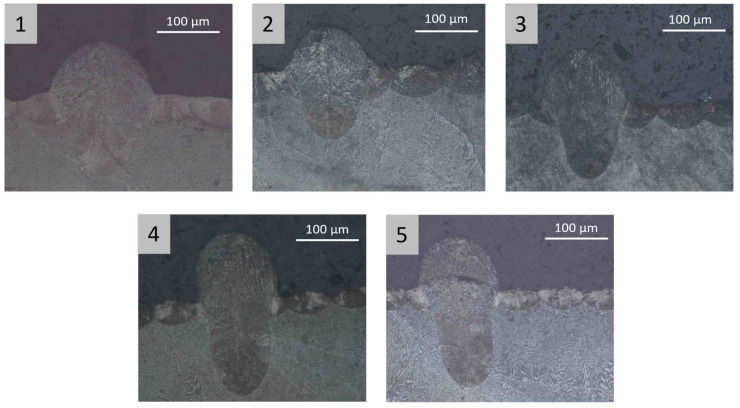
Melt pool shape variation in the specimen tested as a function of tested specimens. The numbers in the gray box indicate the sample as defined in Table 9. Image copyright (2023) Baker Hughes Company—All rights reserved. Used with permission.

The results of the melt pool analysis are summarized in Table 11 and plotted in Figure 20, Figure 21 and Figure 22. Figure 23 shows the images of the melt pool shape in each configuration tested.

## 4. Discussion

### 4.1. Effect of ILCT Variation on Material Microstructure

Upon visual inspection of the specimens after printing, it is possible to state that the sections of the specimens exposed with two seconds of ILCT show a different visual chromatic appearance than the others (Figure 9). The different coloring is probably an indication of the oxidation of the material due to excessive heat and rises in substrate temperature. From this initial assessment, it appears that exposure to two seconds of ILCT could present a criticality on the material having insufficient time to cool down.

The porosity analysis confirms this first indication. The results (Table 6 and Figure 11) show that specimen number six, characterized by an ILCT of two seconds, has the highest porosity value (near 0.1%) with the highest standard deviation (0.038%). Moreover, even from the analysis of Figure 12, tracks of keyhole porosity, commonly related in this process to the excessive amount of heat during printing and heat transfer governed by convection, can be found on that specimen. On the other hand, keyhole defects already begin to appear when the ILCT value reaches six seconds. However, in this case, the defectology present does not cause a macroscopically detectable effect on the specimens, probably due to their small defect dimensions (the maximum defect diameter measured is 58 µm).

By virtue of these results, it is possible to state that, consistent with the material and machine type used to carry out the tests, the ILCT range from 6 to 2 s presents a criticality in terms of densification level.

It is important to note that these ILCT values are difficult to achieve with a single laser printer but easy to achieve with a multi-laser one.

The melt pool analysis confirms this range of ILCT values’ criticality (Table 8, Figure 13, Figure 14 and Figure 15). In fact, a decrease in ILCT leads to an increase in the depth of the melt pool when values lower than six seconds are reached (Table 8). In particular, the melt pool reaches a depth of over 200 microns for the configuration exposed with two seconds of ILCT, causing a remelt of more than three layers on the specimen. This effect is evident by the analysis of Figure 16, in which it is possible to observe the melt pool shape variation due to an ILCT variation from 22 to 2 s (samples 1 and 6) and from 2 to 22 s (samples 6 and 11). It is important to highlight how the melting regime and, consequently, the melt pool shape change as a function of ILCT variation. In particular, samples 1 and 11 are characterized by an almost semi-elliptical shape that indicates that the conductive regime is still present in melt pool formation. On the contrary, the melt pool shape of specimen number 6 is characterized by a very elongated shape peculiar to the keyhole regime.

Relative to the analysis of the effect of overheating and cooling on the specimen by decreasing and increasing the value of ILCT, there are no significant differences in terms of porosity and melt pool shape. For example, specimens 5 and 7, exposed with the same ILCT value, have a similar average melt pool depth (153.9 μm and 156.0 μm, respectively). Similarly, the effect of ILCT on the melt pool width is not significant.

**Figure 24:**
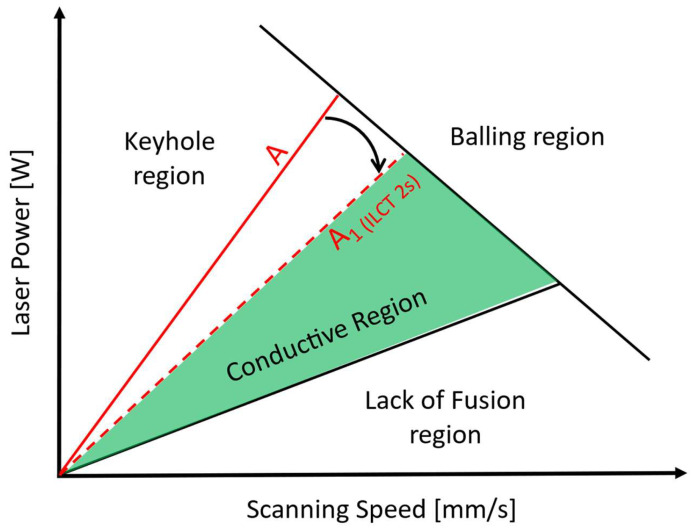
Expansion of keyhole region due to extremely low ILCT shown as a rotation of red line A to red dashed line A1.

By the evaluation of the width/depth ratio (Table 8), it is observed that for specimen number 6, a very low ratio is reached (0.88). In fact, in this case, the melt pool formation is completely governed by convection, while a conduction contribution is still present for the other specimens. So, it is possible to state that very low ILCT values could promote changing the melting regime of the melt pool and the expansion of the keyhole region, as shown in Figure 24.

Moreover, the width/depth ratio results suggest a possible review of the criteria provided by Johnson et al. [46] to calculate the keyhole melting regime as a function of melt pool dimensions.

### 4.2. Effect of Geometries Obstructing Heat Flux on Material Microstructure

The visual inspection of the specimens indicates a different coloring for samples number 4 and 5 (Figure 17). This indication shows the presence of superficial burnishes, probably due to substrate overtemperature. As shown in Table 9 and Figure 18, the results about porosity confirmed the indication of visual inspection with an increasing porosity value and variability for higher range area variation. In particular, specimen number 5 is characterized by the highest porosity value (Avg. = 0.284% and SD = 0.128). These values are about ten times those measured for the reference configuration (specimen number 1). In addition, the evaluation of maximum defect diameter (Table 10 and Figure 19) confirms the significant difference between these configurations.

The melt pool analysis (Table 11) indicated that the exposed area range used for specimens 4 and 5 results is critical. In fact, the melt pool depth value passes from an average value of 153.3 microns for specimen number 1 to 192.8 microns for specimen number 4 and 208.4 microns for specimen 5.

From the analysis of Table 11 and Figure 21, the effect of the exposed area variation on melt pool width is limited.

By analyzing the melt pool shape (Figure 23), it is possible to observe how the melt pool of specimens 1 and 2 has an almost semi-elliptical shape. This means that melt pool formation is not entirely governed by keyhole mode, but the conduction mode is still partially present. On the contrary, the shape of the melt pool for specimens 3, 4 and 5, with a very elongated shape, indicates that the formation of the melt pool is completely governed by keyhole mode. The effect of these phenomena is evident in the progressive reduction of the width/depth ratio as the exposed area range increases (Table 11 and Figure 22).

Under these results, two observations can be pointed out:

- An effect of mitigation of porosity level and reduction of melt pool depth is observed as the surface-to-volume ratio decreases, keeping the printed area for low ILCTs constant. This is evident when comparing the porosity level and melt pool depth measured for specimen number 6 (Table 6 and Table 8) and specimen number 1 (Table 9 and Table 11).
- An effect of amplifying porosity level as the exposed area range increases is registered for an ILCT of 2 s (Table 9). This effect is less pronounced regarding the melt pool depth and width/depth ratio, as can be seen by comparing the results plotted in Table 8 and Table 11.

## 5. Conclusions

The research investigates the effect of ILCT variation during printing on the material’s microstructure (porosity and melt pool analysis) on specimens of nickel-based superalloy Inconel 718. Moreover, the effect of customized geometries that obstruct the heat flux combined with critical ILCT values is investigated.

Considering the obtained results, it is possible to state that extremely low ILCT values can affect the material microstructure regarding densification level and melt pool shape. In particular, an ILCT of less than 6 s introduces criticality in the material microstructure, and a value of 2 s is characterized by widespread keyhole porosity (close to 1‰) and critical melt pool depth (about 200 microns). This effect promotes the expansion of the keyhole region in the printability map.

Moreover, considering geometry obstructing heat flow (low surface-to-volume ratio), exposed with a lower ILCT value (2 s), this effect is strongly amplified, and a very high porosity is measured (about 3‰).

Various processes or design changes can avoid critical ILCT values. In fact, ILCT can be increased by changing geometry, introducing ghost parts during the printing process or using different part orientations. It is important to emphasize that the first solution is often not feasible due to design constraints on component function (dimensions, assembly constraints, et cetera); the second results in an evident loss of productivity; and the last could lead to difficulties in the printing process.

A different approach is used to develop a specific process parameter set for the component sections with critical values for ILCT. In developing this process parameter set, the amplification effect of the keyhole region for short ILCT values must be considered. This approach might be easier to implement and might not have a negative impact on productivity.

An experimental campaign using different materials and boundary conditions, such as L-PBF machines, building platform temperature and process parameter sets, could be interesting to fully characterize this phenomenon and optimize the thermal design of a component in the L-PBF process. Similar results could probably be obtained using other nickel-based superalloys because of their similar printability properties in the L-PBF process.

Moreover, another important aspect to be investigated in future works is the possibility of measuring temperature substrate variation as a function of ILCT values to evaluate the modification of the printability map. This information will be necessary to develop process parameter sets that will be robust to substrate temperature variation. 

## Figures and Tables

**Table 1:** Chemical composition of Inconel 718.

Element	% Weight
C	0.040
Mn	0.08
Si	0.08
P	<0.015
S	0.002
Cr	18.37
Ni	55.37
Co	0.23
Mo	3.04
Nb + Ta	5.34
Ti	0.98
Al	0.5
B	0.004
Ta	0.005
Cu	0.04
Fe	17.80
Ca	<0.01
Mg	<0.01
Pb	0.0001
Bi	0.0001
Se	<0.001
Nb	5.33

**Table 2:** Main properties of Inconel 718 considering mechanical and thermal behaviors.

Elastic Modulus (Gpa)	Yield Strength (Mpa)	Tensile Stress (Mpa)	Strain (%)	Density (kg/m^3^)	Thermal Conductivity (W/mK)
206	1100	1310	23.3	8470	11.2

**Table 3:** Process parameters set used during the test.

Laser Power (W)	390
Scanning Speed (mm/s)	1100
Hatch Distance (mm)	0.09

**Table 4:** Sample dimensions and ILCT levels. The sample code considers the ILCT level of each specimen’s top section, and the letter H or C indicates the heating and cooling phases.

Sample	Sample Code	Radius (mm)	Height (mm)	ILCT Levels (s)
1	22H	10	10	22
2	18H	10	20	22–18
3	14H	10	30	22–18–14
4	10H	10	40	22–18–14–10
5	6H	10	50	22–18–14–10–6
6	2H	10	60	22–18–14–10–6–2
7	6C	10	70	22–18–14–10–6–2–6
8	10C	10	80	22–18–14–10–6–2–6–10
9	14C	10	90	22–18–14–10–6–2–6–10–14
10	18C	10	100	22–18–14–10–6–2–6–10–14–18
11	22C	10	110	22–18–14–10–6–2–6–10–14–18–22

**Table 5:** Specimens’ dimensions (R_min_ is 1.5 mm in all specimens). Sample 1 is a cylinder specimen used as a reference.

Sample	R_max_ (mm)	R_max_ − R_min_ (mm)	Exposed Area Range (mm^2^)	Height (mm)
1	1.5	0.0	7.07–7.07	10
2	2.0	0.5	7.07–12.56	20
3	2.5	1.0	7.07–19.63	30
4	3.0	1.5	7.07–28.26	40
5	3.5	10	7.07–38.46	50

**Table 6:** Porosity analysis results.

Sample	Porosity [%]	
	Avg.	SD
1	0.021	0.019
2	0.023	0.018
3	0.022	0.008
4	0.025	0.011
5	0.059	0.022
6	0.096	0.038
7	0.067	0.024
8	0.028	0.015
9	0.022	0.011
10	0.021	0.009
11	0.021	0.016

**Table 7:** Measures of the maximum defect diameter found in each specimen.

Sample	Maximum Defect Diameter (µm)
2	0.023
3	0.022
4	0.025
5	0.059
6	0.096
7	0.067
8	0.028
9	0.022
10	0.021
11	0.021

**Table 8:** Melt pool analysis results.

Sample	Depth (µm)		Width (µm)		Width/Depth Ratio
	Avg.	SD	Avg.	SD	
1	138.2	11.4	164.6	8.0	1.20
2	142.1	9.9	165.6	7.2	1.20
3	146.5	20.5	167.4	7.9	1.16
4	151.2	11.1	177.1	13.6	1.17
5	153.9	5.0	172.3	11.2	1.12
6	201.7	6.1	172.2	10.4	0.88
7	156.0	7.9	172.6	11.3	1.11
8	152.4	12.3	171.9	9.4	1.13
9	153.1	9.4	166.2	9.3	1.09
10	145.8	13.6	164.0	8.7	1.08
11	146.0	10.3	160.0	15.6	1.10

**Table 9:** Specimens’ dimensions and results of the porosity analysis.

Sample	R_max_ (mm)	R_max_ − R_min_ (mm)	Exposed Area Range (mm^2^)	Porosity (%)	
				Avg.	SD
1	1.5	0.0	7.07–7.07	0.029	0.011
2	2.0	1.5	7.07–12.56	0.054	0.016
3	2.5	1.0	7.07–19.63	0.113	0.044
4	3.0	1.5	7.07–28.26	0.153	0.076
5	3.5	2.0	7.07–38.46	0.284	0.128

**Table 10:** Maximum defect diameter for each specimen.

Sample	Maximum Defect Diameter (µm)
1	21.78
2	34.30
3	58.58
4	71.87
5	96.58

**Table 11:** Melt pool analysis results.

Sample	Depth (µm)		Width (µm)		Width/Depth Ratio
	Avg.	SD	Avg.	SD	
1	120.0	3.8	153.3	5.0	1.27
2	133.6	7.4	159.5	6.1	1.20
3	155.1	6.5	156.3	7.3	1.01
4	190.1	6.2	167.3	8.2	0.86
5	208.4	16.6	166.5	7.7	0.77

## Data Availability

Not applicable.
